# Acute Prostatitis, Terminating in Suppuration, with Complete Urethral and Perineal Fistulæ

**Published:** 1853-03

**Authors:** Napoleon B. Anderson

**Affiliations:** Louisville, Ky.


Art. II.—*Acute Prostatitis, terminating in Suppuration, with complete Urethral and Perineal Fistulæ.* By Napoleon B. Anderson, M. D., Louisville, Ky.

On the 28th of October last, about 3 o'clock, A. M., I was called to see Mr. James Harmon, aged 35 years. He was of the medium stature, athletic, raw-boned, and in possession of an unusually healthy constitution up to the present attack. I found him lying upon his back, with the knees drawn forward toward the abdomen; a high fever; flushed countenance; pulse one hundred and thirty, and hard; with an uneasy pain and scalding sensation in the region of the neck of the bladder; frequent and ineffectual desire to micturate; pain over the hypogastric region, with great distention of the bladder; thirst intense; tongue coated with a thick brown covering; eyes congested, with great pain over the orbits. I learned from him that he had had gonnorrhea, the discharge of which had been checked several days previous, by a strong injection of nitrate of silver; that he had not urinated for twenty hours, nor been purged for the period of three days. The finger introduced into the rectum, and gentle pressure upon the prostate gland, imparted the most excruciating pain, which extended down the inner portion of the thighs, to the lumbar and hypogastric regions, the glans-penis and scrotum.

He was bled from the arm freely, and the urine drawn off. The catheter was introduced with great difficulty, owing to the swollen condition of the prostate gland. A purgative was ordered, as follows:

℞.—Sulph. Magnesia, ℥ ij.

Ant. et Pot. Tart, gr. j.

Aquæ Menth., ℥ iv. Mix.

A table spoonful of which was given every hour, until purgative effects were produced.

Eight o'clock, A. M.—Has had several copious alvine evacuations; fever still high, with a dry hot skin; countenance flushed ; pulse one hundred and thirty, full and corded ; bladder distended, with considerable tenderness over the pubic region, extending down the thighs and to the perineum, the latter of which being considerably swollen.

He was bled again to syncope ; ten leeches were applied to the perineum, and cups to the hypogastric region, followed by hot hop fomentations. He was placed on tartar emetic and Dover's powder. His urine, on this occasion, was drawn off with the greatest difficulty, causing the most excruicating pain; it was high colored, thick and ropy. In fact, the catheter could not have been introduced at all, but by the aid of the finger carried into the rectum, and pressed gently upon the prostate gland, with a tedious, yet gentle pressure of the instrument against the parts, which lasted for near half an hour.

Six o'clock, P. M. — Fever abated ; pulse soft and one hundred ; perspiring profusely ; has had three evacuations from the bowels ; bladder again distended, though not so tense and painful as on the previous occasions, yet a constant effort and painful desire to urinate continues ; the perineum much swollen, and very sensative to the touch. Ordered a hot bath ; the urine to be drawn off, which continued thick and tinged with blood ; an enema of sixty drops of laudanum in two ounces of starch water warmed, was ordered into the rectum, and repeated at intervals of three to four hours. Calomel and Dover's powder continued, with the hot hop fomentations to the parts.

29th — Seven o'clock, A. M. — Has rested well during the night ; slight fever, with a moist skin ; pulse one hundred and soft ; tongue nearly clean, with red tip and edges ; bladder distended ; perinaeum greatly swollen and very painful, attended by a dull aching, throbbing and shooting pain, extending from the neck of the bladder and prostate gland to the glans-penis and scrotum, the latter of which is much swollen.

Ten additional leeches were applied to the perineum, followed by hot hop fomentations to the parts. The urine was again drawn off, which continued bloody, with the existence of an urethral discharge in appearance like that of gonnorrhea. The calomel and Dover's powder were continued.

30—Eight, A. M.—No fever; skin moist; pulse one hundred, and soft; tongue clean, with the edges continuing red; bladder distended and painful; the perineum greatly swollen and extremely tender. The urine was drawn off with much pain and difficulty, remaining thick and bloody. There existed a copious discharge from the urethra, of bloody pus, attended by severe pulsatory pain through the prostate gland and perineum, with scalding of the urethra.

The calomel and Dover's powder were continued; ten leeches again applied to the perineum, followed by the fomentations.

Six o'clock, P. M. — Symptoms materially changed from yesterday. On introducing the finger into the rectum, the prostate gland was found materially enlarged, and very sensitive; which, together with the extent of the œdematous perineum—the discharge from the urethra — the aching, throbbing local pain, with a sense of great weight at the neck of the bladder, and the constant desire to urinate — the scalding sensation in the urethra — the feeling of fulness, as of a foreign body in the rectum, with more or less uneasiness in all the associated organs — the dryness of the skin, with acceleration of fever — increased flush of the countenance — with a circumscribed portion of the perineum more tender and dependent than the surrounding parts — led to the surmise of an abcess.

The calomel and Dover's powder were continued, and the bowels moved by an enema. A hot emollient poultice was applied to the perineum, and renewed by a fresh one every three hours.

31st — Nine o'clock, A. M. — The fever is much higher than yesterday — pulse one hundred and thirty, and hard — tongue dry and parched — thirst intense — bladder full and painful — perineum much swollen and slight fluctuation in the region of the gland — a copious, thick and bloody discharge from the urethra, mixed with pus.

The urine was drawn off, and the dependent portion of the perineum laid open with the bistoury, from which a large portion of thick, dark bloody matter escaped.

The poultices were renewed, and alternate doses of Dover's powder and a solution of the acetate of ammonia, followed by demulcent drinks were continued. Anodyne injections were ordered into the rectum, and repeated at such intervals as the symptoms called for.

1st Nov. — Nine o'clock, A. M .— He has rested well the previous night — has urinated freely for the first time since the outset of the attack; it contains a mixture of pus, being thick, ropy and bloody. The quantity of matter that has escaped from the opening made into the perineum has been large, attended by small quantities of urine. The treatment of the previous day was continued.

2d—Ten o'clock, A. M.—No excitement exists whatever — has urinated freely — a considerable discharge from the perineum, attended by a much larger quantity of urine than on yesterday. All medicine suspended, and the patient put upon a nourishing diet, with demulcent drinks. The poultices were continued to the perineum.

3d — Ten o'clock, A. M. — Improving fast — passed the urine freely, which is mixed with less blood, but an augmentation of pus. The perineum not so much swollen, with a decrease in the discharge of matter, but attended by a larger flow of urine.

The warm water dressings were ordered, and the urine to be drawn off at regular intervals, by the use of the catheter. Tepid castile soap-suds were injected every four hours into the perineal fistula.

This course of treatment was continued for the period of ten days, when the urine was suffered to flow through its accustomed channel; the perineal discharge was suspended, and the organs speedily resumed their wonted functions.

The continuance, for some weeks afterwards, of the gonorrheal discharge, called for the use of balsam copaiba, rest of the organs, cleanliness, and a light diet, which, when put in requisition, speedily terminated in a perfect recovery.

LOUISVILLE, February, 1853.

